# Supplementary material for: Adaptation and validation of the Polish version of the Beliefs about Medicines Questionnaire among cardiovascular patients and medical students
Source: PLoS One. 2020 Apr 13;15(4):e0230131. doi: 10.1371/journal.pone.0230131 (PMC7153860; doi:10.1371/journal.pone.0230131)
Supplement: S1 File — (DOCX) [file pone.0230131.s001.docx]

**Supporting Information 1**

**Understanding the BMQ-PL**

Three major notions, which were identified as a result of semi-structured interviews with cardiovascular patients regarding the BMQ-PL content:

**1) The *General* beliefs about medicines are largely formed through patients’ personal experience with the medications.** Patients refer to their own history, while being asked to state in their own words how they understand each *General* statement. For example, a patient asked to describe what the statement “Most medicines are addictive” means, replied personally “I can become addicted, I will not be able to live without medicine, I will have to take it all the time”. Another example is: a patient asked to describe what kind of harm is mentioned in the statement “Medicines do more harm than good”, replied spontaneously at first “Hmm, I really don’t know, I didn’t experience any harm from medicines.”

**2) The respondents have problems assessing whether a given *General* statement concerns the aspects of *Overuse* or *Harm*.** Patients tended to attribute negative consequences of using medications inseparably to both the pattern of their use (*Overuse*) and intrinsic harm (*Harm*). For example, a patient who was asked about the meaning of the statement “Most medicines are addictive”, started her response with: “What too much (*Overuse*) is not healthy – and may be damaging (*Harm*)…”. Patients could also explain that a *General-Overuse* statement, eg “Natural remedies are safer than medicines”, means that “the natural, herbal products are safe, have no side effects (no intrinsic *Harm*)”. Moreover, at the end of the interview, each patient was asked to simply classify each *General* statement into the category *Overuse* or *Harm*, and each *Specific* statement into the category *Necessity* or *Concern*. As a result, 25 out of 48 *General* patient-statements (52.1%) were mistakenly attributed to the opposite category, whereas the mistakes in classification of *Specific* statements were much less common (11 out of 60 patient-statements, ie 18.3%), χ^2^(1)=13.7, *p*=0.0002. The statement with the highest number of incorrect classifications (*Harm* instead of *Overuse*) was “Natural remedies are safer than medicines” (5 out of 6).

**3) The statement *My medicines are a mystery to me* caused reflection and hesitation among patients.** The word *mystery* was in fact too “mysterious” to many respondents. However, most patients correctly classified this statement to the *Concern* category and indicted that they associate it with the uncertainty of medication benefits and possibility of risk.
