# Supplementary material for: Adaptation and validation of the Polish version of the Beliefs about Medicines Questionnaire among cardiovascular patients and medical students
Source: PLoS One. 2020 Apr 13;15(4):e0230131. doi: 10.1371/journal.pone.0230131 (PMC7153860; doi:10.1371/journal.pone.0230131)
Supplement: S2 File — (DOCX) [file pone.0230131.s002.docx]

**Supporting Information 2**

**Measurement invariance between the tested groups of patients**

The criteria for rejection of null hypothesis about invariance between the compared group of patients were:

- the statistically significant test for Δχ^2^ of the models (*p*<0.05) and
- ΔRMSEA equal or above 0.01 and
- ΔCFI equal or below -0.01 (Fall et al. 2014).

**Table. Measurement invariance between cardiovascular in- and outpatients.**

| Model number | Description | Chi2 (df) | RMSEA (90% CI) | CFI | Model compared | Δχ^2^ (Δdf) | *P*-value | ΔRMSEA | ΔCFI | Rejection of the  null hypothesis  of invariance |
| --- | --- | --- | --- | --- | --- | --- | --- | --- | --- | --- |
| 1 | Full configural invariance | 373.574 (258) | 0.068 (0.052-0.082) | 0.873 |  |  |  |  |  |  |
| 2 | Full metric invariance | 398.704 (276) | 0.067 (0.052-0.082) | 0.866 | 1 | 25.13 (18) | 0.12 | -0.001 | -0.007 | No |
| 3 | Full scalar invariance | 457.466 (294) | 0.075 (0.062-0.089) | 0.829 | 2 | 58.76 (18) | <0.0001 | 0.008 | -0.037 | Yes |
| 3.1 | Partial scalar invariance (SN constrain) | 407.453 (281) | 0.068 (0.053-0.082) | 0.862 | 2 | 8.75 (5) | 0.12 | 0.001 | -0.004 | No |
| 3.2 | Partial scalar invariance (SC constrain) | 414.984 (281) | 0.070 (0.055-0.084) | 0.856 | 2 | 16.28 (5) | 0.0061 | 0.003 | -0.010 | Yes |
| 3.3 | Partial scalar invariance (GO constrain) | 406.720 (280) | 0.068 (0.053-0.082) | 0.861 | 2 | 8.02 (4) | 0.091 | 0.001 | -0.005 | No |
| 3.4 | Partial scalar invariance (GH constrain) | 420.413 (280) | 0.072 (0.057-0.085) | 0.849 | 2 | 21.71 (4) | 0.0002 | 0.005 | -0.017 | Yes |
| 3.5 | Partial scalar invariance (SN and GO constrain) | 415.370 (285) | 0.068 (0.054-0.082) | 0.858 | 2 | 16.67 (9) | 0.054 | 0.001 | -0.008 | No |
| 4 | Full residual variance invariance | 524.233 (303) | 0.086 (0.074-0.099) | 0.775 | 3.5 | 108.86 (18) | <0.0001 | 0.018 | -0.083 | Yes |
| 4.1 | Partial residual variance invariance (SN constrain) | 482.131 (290) | 0.082 (0.069-0.095) | 0.797 | 3.5 | 66.76 (5) | <0.0001 | 0.014 | -0.061 | Yes |
| 4.2 | Partial residual variance invariance (SC constrain) | 426.320 (290) | 0.069 (0.055-0.083) | 0.852 | 3.5 | 10.95 (5) | 0.052 | 0.001 | -0.006 | No |
| 4.3 | Partial residual variance invariance (GO constrain) | 429.199 (289) | 0.070 (0.056-0.084) | 0.850 | 3.5 | 13.83 (4) | 0.0079 | 0.002 | -0.008 | Yes |
| 4.4 | Partial residual variance invariance (GH constrain) | 433.177 (289) | 0.071 (0.057-0.085) | 0.845 | 3.5 | 17.81 (4) | 0.0013 | 0.003 | -0.013 | Yes |

SN – *Specific-Necessity* subscale

SC – *Specific-Concerns* subscale

GO – *General-Overuse* subscale

GH – *General-Harm* subscale

χ^2^ – chi-square statistics, df – degrees of freedom, CI – confidence intervals, RMSEA – Root Mean Square Error of Approximation, CFI – Comparative Fit Index

**Table. Measurement invariance between cardiovascular inpatients and medical students**

| Model number | Description | Chi2 (df) | RMSEA (90% CI) | CFI | Model compared | Δχ^2^ (Δdf) | *P*-value | ΔRMSEA | ΔCFI | Rejection of the  null hypothesis  of invariance |
| --- | --- | --- | --- | --- | --- | --- | --- | --- | --- | --- |
| 1 | Full configural invariance | 398.936 (258) | 0.073 (0.059-0.087) | 0.849 |  |  |  |  |  |  |
| 2 | Full metric invariance | 455.991 (276) | 0.080 (0.067-0.093) | 0.807 | 1 | 57.06 (18) | 0.0001 | 0.007 | -0.042 | Yes |
| 2.1 | Partial metric invariance (SN constrain) | 428.503 (263) | 0.078 (0.065-0.092) | 0.823 | 1 | 29.57 (5) | <0.0001 | 0.005 | -0.026 | Yes |
| 2.2 | Partial metric invariance (SC constrain) | 407.586 (263) | 0.073 (0.059-0.087) | 0.845 | 1 | 8.65 (5) | 0.12 | 0.000 | -0.004 | No |
| 2.3 | Partial metric invariance (GO constrain) | 410.859 (262) | 0.075 (0.060-0.088) | 0.840 | 1 | 11.92 (4) | 0.018 | 0.002 | -0.009 | Yes |
| 2.4 | Partial metric invariance (GH constrain) | 405.317 (262) | 0.073 (0.059-0.087) | 0.846 | 1 | 6.38 (4) | 0.17 | 0.000 | -0.003 | No |
| 2.5 | Partial metric invariance (SC and GH constrain) | 413.747 (267) | 0.073 (0.059-0.087) | 0.843 | 1 | 14.81 (9) | 0.096 | 0.000 | -0.006 | No |
| 3 | Full scalar invariance | 685.178 (285) | 0.117 (0.106-0.128) | 0.731 | 2.5 | 271.43 (18) | <0.0001 | 0.044 | -0.112 | Yes |
| 3.1 | Partial scalar invariance (SN constrain) | 492.050 (272) | 0.089 (0.076-0.101) | 0.792 | 2.5 | 78.30 (5) | <0.0001 | 0.016 | -0.051 | Yes |
| 3.2 | Partial scalar invariance (SC constrain) | 568.28 (272) | 0.103 (0.091-0.115) | 0.746 | 2.5 | 154.53 (5) | <0.0001 | 0.030 | -0.097 | Yes |
| 3.3 | Partial scalar invariance (GO constrain) | 431.571 (271) | 0.076 (0.062-0.089) | 0.830 | 2.5 | 17.82 (4) | 0.0013 | 0.003 | -0.013 | Yes |
| 3.4 | Partial scalar invariance (GH constrain) | 518.785 (271) | 0.094 (0.082-0.107) | 0.778 | 2.5 | 105.04 (4) | <0.0001 | 0.021 | -0.065 | Yes |

SN – *Specific-Necessity* subscale

SC – *Specific-Concerns* subscale

GO – *General-Overuse* subscale

GH – *General-Harm* subscale

χ^2^ – chi-square statistics, df – degrees of freedom, CI – confidence intervals, RMSEA – Root Mean Square Error of Approximation, CFI – Comparative Fit Index

**Table. Measurement invariance between cardiovascular outpatients and medical students**

| Model number | Description | Chi2 (df) | RMSEA (90% CI) | CFI | Model compared | Δχ^2^ (Δdf) | *P*-value | ΔRMSEA | ΔCFI | Rejection of the  null hypothesis  of invariance |
| --- | --- | --- | --- | --- | --- | --- | --- | --- | --- | --- |
| 1 | Full configural invariance | 409.191 (258) | 0.077 (0.062-0.091) | 0.824 |  |  |  |  |  |  |
| 2 | Full metric invariance | 433.663 (276) | 0.076 (0.062-0.089) | 0.816 | 1 | 24.47 (18) | 0.14 | -0.001 | -0.008 | No |
| 3 | Full scalar invariance | 626.522 (294) | 0.107 (0.095-0.118) | 0.712 | 2 | 192.86 (18) | <0.0001 | 0.031 | -0.104 | Yes |
| 3.1 | Partial scalar invariance (SN constrain) | 484.799 (281) | 0.085 (0.072-0.098) | 0.781 | 2 | 51.14 (5) | <0.0001 | 0.009 | -0.035 | Yes |
| 3.2 | Partial scalar invariance (SC constrain) | 535.995 (281) | 0.096 (0.083-0.108) | 0.739 | 2 | 102.33 (5) | <0.0001 | 0.020 | -0.077 | Yes |
| 3.3 | Partial scalar invariance (GO constrain) | 457.891 (280) | 0.080 (0.067-0.093) | 0.796 | 2 | 24.23 (4) | 0.0001 | 0.004 | -0.020 | Yes |
| 3.4 | Partial scalar invariance (GH constrain) | 486.866 (280) | 0.086 (0.073-0.099) | 0.780 | 2 | 53.20 (4) | <0.0001 | 0.010 | -0.036 | Yes |

SN – *Specific-Necessity* subscale

SC – *Specific-Concerns* subscale

GO – *General-Overuse* subscale

GH – *General-Harm* subscale

χ^2^ – chi-square statistics, df – degrees of freedom, CI – confidence intervals, RMSEA – Root Mean Square Error of Approximation, CFI – Comparative Fit Index

**Reference**

Fall E, Gauchet A, Izaute M, Horne R, Chakroun N. Validation of the French version of the Beliefs about Medicines Questionnaire (BMQ) among diabetes and HIV patients. *Eur Rev Appl Psychol. 2014*;64:335-343.
