# Supplementary material for: Adaptation and validation of the Polish version of the Beliefs about Medicines Questionnaire among cardiovascular patients and medical students
Source: PLoS One. 2020 Apr 13;15(4):e0230131. doi: 10.1371/journal.pone.0230131 (PMC7153860; doi:10.1371/journal.pone.0230131)
Supplement: S3 File — (DOCX) [file pone.0230131.s003.docx]

**Supporting Information 3**

**Further adaptation of the BMQ-General for the group of medically-educated people**

A group of 80 medical students (59 females, mean age 23.4 ± 2.1 years) completed the electronic survey to attribute each *Specific* BMQ-PL statement to the category of *Necessity*, *Concerns* or *None of the indicated* and each *General* BMQ-PL statement to the category of *Overuse*, *Harm* or *None of the indicated*. The results are presented in the Table below. The statement S6 (*My medicines are a mystery to me*), although correctly attributed to the *Concerns* category, exhibited a considerable number of “None of the indicated” answers. This could be explained by its “mysterious” meaning, as revealed by the semi-structured interviews with the patients. The statement G4 (*Natural remedies are safer than medicines*) was predominantly classified to the category of *Harm*.

**Table. Classification of the BMQ-PL statements**

a) BMQ-PL *Specific*

| Statement | Which facet does this statement represent? | | |
| --- | --- | --- | --- |
|  | Necessity | Concerns | None of the indicated |
| Necessity | | | |
| S1 | **72 (90.0%)** | 1 (1.2%) | 7 (8.8%) |
| S3 | **68 (85.0%)** | 2 (2.5%) | 10 (12.5%) |
| S4 | **70 (87.5%)** | 2 (2.5%) | 8 (10.0%) |
| S7 | **53 (66.2%)** | 12 (15.0%) | 15 (18.8%) |
| S10 | **74 (92.5%)** | 3 (3.8%) | 3 (3.8%) |
| Concerns | | | |
| S2 | 7 (8.8%) | **66 (82.5%)** | 7 (8.8%) |
| S5 | 2 (2.5%) | **72 (90.0%)** | 6 (7.5%) |
| S6 | 1 (1.2%) | **43 (53.8%)** | 36 (45.0%) |
| S8 | 3 (3.8%) | **57 (71.2%)** | 20 (25.0%) |
| S9 | 2 (2.5%) | **72 (90.0%)** | 6 (7.5%) |

b) BMQ-PL *General*

| Statement | Which facet does this statement represent? | | |
| --- | --- | --- | --- |
|  | Overuse | Harm | None of the indicated |
| Overuse | | | |
| G1 | **68 (85.0%)** | 11 (13.8%) | 1 (1.2%) |
| G4 | **4 (5.0%)** | 59 (73.8%) | 17 (21.2%) |
| G7 | **61 (76.2%)** | 8 (10.0%) | 11 (13.8%) |
| G8 | **61 (76.2%)** | 3 (3.8%) | 16 (20.0%) |
| Harm | | | |
| G2 | 23 (28.8%) | **49 (61.2%)** | 8 (10.0%) |
| G3 | 17 (21.2%) | **56 (70.0%)** | 7 (8.8%) |
| G5 | 7 (8.8%) | **66 (82.5%)** | 7 (8.8%) |
| G6 | 5 (6.2%) | **66 (82.5%)** | 8 (10.0%) |

S1-10 – subsequent *Specific* items, G1-8 – subsequent *General* items

Considering unfavorable results of exploratory factor analysis of the *General* scale of the BMQ-PL in medical students (Table 3 in the main body of manuscript), and the scientific interest in this scale, manifested in substantial number of publications using BMQ-General among healthcare professionals, we decided to further adapt this scale only. The semi-structured interviews performed with six medical students and young medical doctors (two female, mean age 27.3 ± 5.0 years) revealed that general beliefs about medicines are typically formed through academic knowledge and clinical experience. Thus, the respondents perceived the statements through the prism of medical knowledge and current therapeutic recommendations. Particularly, although the majority of respondents did not agree with statement G6 (*All medicines are poisons*), those who did, attributed the meaning of the sentence to the famous Paracelsus dictum “…all things are poison and nothing is without poison…”. Consequently, this statement did not examine the beliefs about medicines being harmful, but rather how strict the respondent adheres to the formal toxicological definition. This statement was excluded from the questionnaire. On the other hand, basing on the interviews, the results about classification of the statements and exploratory factor analysis, the statement G4 (*Natural remedies are safer than medicines*) was transferred to the category *General-Harm*. In this form, the scale (BMQ-PL-General-Med) exhibited satisfactory face validity as assessed by the interviewees. Exploratory factor analysis suggested 2-factor solution (based on the eigenvalue-more-than-one criterion and the scree-plot analysis) explaining 55.2% of variance with satisfactorily high loadings (>0.6) and negligible cross-loadings (<0.3).

Finally, the BMQ-PL-General-Med scale was tested with confirmatory factor analysis in the group of medical students taking medications chronically (n=107), and externally validated in the testing dataset, ie the students who declared not taking medications chronically (less than 90 days a year), who completed BMQ-General scale only (n=296: 162 females, mean age 23.2 ± 1.5 years). The model fit was good in the validation dataset, especially in the group after exclusion of dental students, whose primary therapeutic means are not medicines. It should be noted that the model fit parameter estimates may be not fully accurate due to low statistical power of the analysis. Internal consistency of the scales was satisfactory for the BMQ-PL-General-Med (see Table below).

**Table. Confirmatory factor analysis model fit parameters and internal consistency estimates for the newly proposed BMQ-PL-General-Med scale**

|  | | | Training dataset (n=107) | | Validation dataset (n=296) | Validation dataset (n=260)* |
| --- | --- | --- | --- | --- | --- | --- |
|  |  |  | Original 8-item BMQ-General | Modified 7-item BMQ-General | | |
| χ^2^ (df) | | | 35.7 (19) | 14.6 (13) | 30.4 (13) | 21.2 (13) |
| *P*-value | | | 0.011 | 0.33 | 0.0041 | 0.070 |
| χ^2^/df | | | 1.88 | 1.13 | 2.34 | 1.63 |
| RMSEA (90% CI) | | | 0.093 (0.045-0.139) | 0.034 (0-0.105) | 0.071 (0.041-0.103) | 0.052 (0-0.089) |
| TLI | | | 0.798 | 0.976 | 0.925 | 0.956 |
| CFI/PCFI | | | 0.864 / 0.518 | 0.986 / 0.555 | 0.953 / 0.571 | 0.973 / 0.579 |
| SRMR | | | 0.096 | 0.058 | 0.047 | 0.041 |
| Internal consistency | Cronbach’s alpha | *General-Overuse* | 0.66 | 0.74 | 0.69 | 0.68 |
|  |  | *General-Harm* | 0.42 | 0.58 | 0.65 | 0.63 |
|  | McDonald’s omega | | 0.76 | 0.80 | 0.81 | 0.80 |
| Statistical power | | | 0.249 | 0.202 | 0.482 | 0.433 |

* after exclusion of dental students

χ^2^ – chi-square statistics

df – degrees of freedom

CI – confidence intervals

RMSEA – Root Mean Square Error of Approximation. The strict cut-off point estimate close to 0.06 with a lower 90% CI limit close to 0 and the upper limit less than 0.08 is currently considered “a good fit”. In the past, however, the recommendations were less strict: a point estimate below 0.08 was considered “a good fit”, whereas between 0.08 to 0.10 “a mediocre fit”.

TLI – Tucker-Lewis index or Non-Normed Fit Index. Values at least 0.95 are preferred, but values as low as 0.80 were also acceptable.

CFI – Comparative Fit Index. Values at least 0.95 are presently recognized as indicative of “a good fit”, but the limit of 0.90 was proposed in the past.

PCFI – Parsimony-corrected Comparative Fit Index. While no threshold levels have been recommended for parsimony-corrected indices, it is suggested that the values of at least 0.50 or, even better, 0.60 should be obtained.

SRMR – standardized root mean square residual. Values less than 0.05 represent “a good fit”, however, values as high as 0.08 are deemed “acceptable”.
