# Supplementary material for: Adaptation and validation of the Polish version of the Beliefs about Medicines Questionnaire among cardiovascular patients and medical students
Source: PLoS One. 2020 Apr 13;15(4):e0230131. doi: 10.1371/journal.pone.0230131 (PMC7153860; doi:10.1371/journal.pone.0230131)
Supplement: S1 Table — Each row presents results of association between Variable 1 and Variable 2 in a given group of patients (PDF) [file pone.0230131.s004.pdf]

S1 Table. Pearson's r and Spearman's rho coefficients for the associations. Each row presents results of association between Variable 1 and Variable 2 in a given group of patients

Group	Variable 1	Variable 2	Parametric test		Non-parametric test		Is statistical conclusion consistent?
			Pearson's r	*P*-value	Spearman's rho	*P*-value	
Association between the BMQ-PL subscales							
Inp.	SN	SC	0.0143	0.89	0.0595	0.55	Yes
Inp.	SN	GO	-0.2822	0.0041	-0.2517	0.011	Yes
Inp.	SN	GH	-0.2793	0.0045	-0.1700	0.088	No
Inp.	SC	GO	0.4404	<0.0001	0.3710	0.0001	Yes
Inp.	SC	GH	0.3890	<0.0001	0.3716	0.0001	Yes
Inp.	GO	GH	0.5301	<0.0001	0.4579	<0.0001	Yes
Outp.	SN	SC	0.0751	0.47	0.0453	0.66	Yes
Outp.	SN	GO	-0.0903	0.38	-0.0711	0.49	Yes
Outp.	SN	GH	-0.0864	0.40	-0.1542	0.13	Yes
Outp.	SC	GO	0.3965	<0.0001	0.3776	0.0001	Yes
Outp.	SC	GH	0.4441	<0.0001	0.4090	<0.0001	Yes
Outp.	GO	GH	0.6109	<0.0001	0.5388	<0.0001	Yes
Stud.	SN	SC	0.1753	0.071	0.1523	0.12	Yes
Stud.	SN	GO	-0.0525	0.59	-0.0685	0.49	Yes
Stud.	SN	GH	-0.1321	0.18	-0.1034	0.29	Yes
Stud.	SC	GO	0.3051	0.0015	0.3349	0.0004	Yes
Stud.	SC	GH	0.2821	0.0034	0.2519	0.0092	Yes
Stud.	GO	GH	0.4469	<0.0001	0.4370	<0.0001	Yes
Raw association of the BMQ-PL subscales with self-reported measure of drug adherence							
Inp.	ARMS	SN	-0.0376	0.71	-0.1299	0.19	Yes
Inp.	ARMS	SC	-0.2062	0.038	-0.1850	0.063	No
Inp.	ARMS	SN-SC	0.1351	0.18	0.0695	0.49	Yes
Inp.	ARMS	GO	0.0764	0.45	0.1215	0.22	Yes
Inp.	ARMS	GH	-0.1101	0.27	-0.0005	1.0	Yes
Outp.	ARMS	SN	0.2871	0.0053	0.2498	0.016	Yes
Outp.	ARMS	SC	-0.2370	0.024	-0.2145	0.041	Yes
Outp.	ARMS	SN-SC	0.3965	0.0001	0.4135	<0.0001	Yes
Outp.	ARMS	GO	-0.2664	0.0091	-0.3033	0.0028	Yes
Outp.	ARMS	GH	-0.3009	0.0030	-0.2878	0.0047	Yes
Association between the BMQ-PL subscales and the number of medications used							
All	Med.	SN	0.3312	<0.0001	0.3137	<0.0001	Yes
All	Med.	SC	0.3217	<0.0001	0.3137	<0.0001	Yes
All	Med.	GO	0.0088	0.88	0.0134	0.82	Yes
All	Med.	GH	0.1887	0.0012	0.1975	0.0007	Yes

Inp. – Inpatients
Outp. – Outpatients
Stud. – Medical students
ARMS – self-reported measure of drug adherence assessed with Polish version of Adherence to Refills and Medications Scale
SN – *Specific-Necessity* subscale
SC – *Specific-Concerns* subscale
SN-SC – difference between *Specific-Necessity* and *Specific-Concerns* subscales
GO – *General-Overuse* subscale
GH – *General-Harm* subscale
