# Supplementary figures and images for: Adaptation and validation of the Polish version of the Beliefs about Medicines Questionnaire among cardiovascular patients and medical students
Source: PLoS One. 2020 Apr 13;15(4):e0230131. doi: 10.1371/journal.pone.0230131 (PMC7153860; doi:10.1371/journal.pone.0230131)

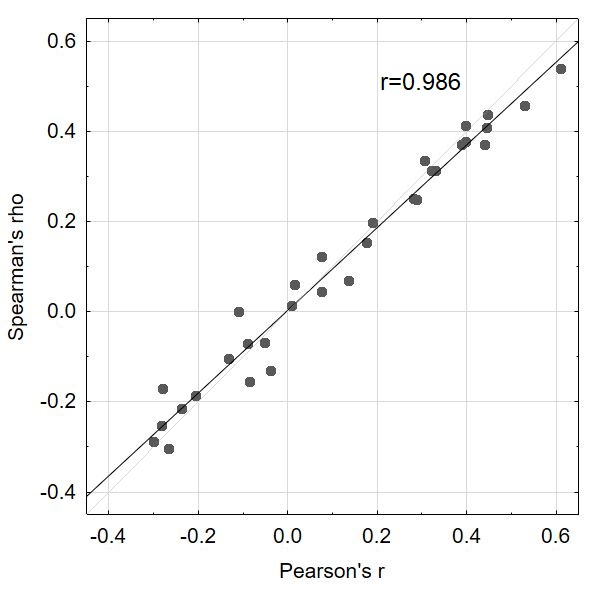

Supplement: S1 Fig — The points represent corresponding coefficients given in S1 Table. (TIF) [file pone.0230131.s005.tif]
